# Biological Evaluation of 3-Azaspiro[Bicyclo[3.1.0]Hexane-2,5′-Pyrimidines] as Potential Antitumor Agents

**DOI:** 10.3390/ijms231810759

**Published:** 2022-09-15

**Authors:** Stanislav V. Shmakov, Diana K. Latypova, Tatiana V. Shmakova, Artem A. Rubinshtein, Mark V. Chukin, Sergei G. Zhuravskii, Nickolay A. Knyazev, Alexander V. Stepakov, Michael M. Galagudza, Vitali M. Boitsov

**Affiliations:** 1 Laboratory of Nanobiotechnologies, Saint-Petersburg National Research Academic University of the Russian Academy of Sciences, 194021 Saint-Petersburg, Russia; 2 Institute of Experimental Medicine, Almazov National Medical Research Centre, Ministry of Health of the Russian Federation, 194156 Saint-Petersburg, Russia; 3 Scientific Research Center, Pavlov First Saint Petersburg State Medical University, 197022 Saint-Petersburg, Russia; 4 Institute of Cytology, Russian Academy of Sciences, 194064 Saint-Petersburg, Russia; 5 Saint-Petersburg Clinical Scientific and Practical Center for Specialized Types of Medical Care (Oncological), 197758 Saint-Petersburg, Russia; 6 Department of Chemistry, Saint-Petersburg State University, 199034 Saint-Petersburg, Russia; 7 Department of Organic Chemistry, Saint Petersburg State Institute of Technology, 190013 Saint-Petersburg, Russia

**Keywords:** 3-azaspiro[bicyclo[3.1.0]hexane-2,5′-pyrimidines], alloxan-derived azomethine ylide, cyclopropenes, cancer cell lines (K-562; HeLa; Jurkat; CT26; Vero), antiproliferative activity, cell motility, morphological changes (cytoskeleton), cell cycle, cell death, in vitro and in vivo activity

## Abstract

A series of heterocyclic compounds containing spirofused barbiturate and 3-azabicyclo[3.1.0]hexane frameworks have been studied as potential antitumor agents. Antiproliferative activity of products was screened in human erythroleukemia (K562), T lymphocyte (Jurkat), and cervical carcinoma (HeLa) as well as mouse colon carcinoma (CT26) and African green monkey kidney epithelial (Vero) cell lines. The most effective among the screened compounds show IC_50_ in the range from 4.2 to 24.1 μM for all tested cell lines. The screened compounds have demonstrated a significant effect of the distribution of HeLa and CT26 cells across the cell cycle stage, with accumulation of cells in SubG1 phase and induced apoptosis. It was found, using a confocal microscopy, that actin filaments disappeared and granular actin was distributed diffusely in the cytoplasm of up to 90% of HeLa cells and up to 64% of CT26 cells after treatment with tested 3-azaspiro[bicyclo [3.1.0]hexane-2,5′-pyrimidines]. We discovered that the number of HeLa cells with filopodium-like membrane protrusions was reduced significantly (from 91% in control cells to 35%) after treatment with the most active compounds. A decrease in cell motility was also noticed. Preliminary in vivo experiments on the impact of the studied compounds on the dynamics of CT26 tumor growth in Balb/C mice were also performed.

## 1. Introduction

Cancer is one of the most frequent health problems worldwide and, after cardiovascular diseases, is the second-leading cause of mortality. The development of drug resistance and the severe side-effects of chemotherapeutic agents reduce the clinical efficacy of the currently used anticancer drugs and treatments, which may lead to cancer treatment failure and an increased likelihood of cancer recurrence and metastasis. The development of cytostatic agents still remains an essential task for cancer therapy, despite the growing application of targeted drugs and methods of immunotherapy. At the same time, the development of drug resistance requires the generation of new chemical entities that are not just “classical” drugs’ derivatives but are arising from new natural compounds. Therefore, special attention is paid to studying biological action mechanisms of new low-molecular biologically active compounds and to optimizing their structure by functionally oriented (molecular) design, in order to develop drugs for targeted therapy.

Natural products or artificial compounds created on the basis of natural products are still excellent sources of new candidate drugs. To date, many of the most successful applicable anticancer drugs either are natural origin compounds or are created on the basis of such compounds [1,2]. Most frequently, these are complex structure compounds that can be produced as a result of multistage synthesis only. Recent achievements and improvements in the complex-fused heterocyclic system’s synthesis led to a substantial growth in interest in the development of efficient methods for the synthesizing of various structural analogues and derivatives of these compounds as potential drugs or biological probes [3,4]. So, azabicyclohexane, indenoquinoxaline, pyrrolizine, pyrroloisoquinoline, and oxindole units represent a heterocyclic motif, which forms the core of a huge alkaloid natural products family with strong bioactivity profiles. Thus, azabicyclo[3.1.0]hexane is an important core structure found in many natural compounds [5,6,7], which are parts of pharmaceuticals [8,9,10] as well as valuable intermediate products [11,12,13]. Note that compounds comprising azabicyclo[3.1.0]hexane moiety are inhibitors of histone deacetylase [14], antagonists of morphine-induced antinociception [15], and antagonists of opioid receptors [10,12,16] and the dopamine D3 receptor [17]; additionally, they show antibacterial activity [18,19]. Barbiturates (barbituric acid derivatives) are widely known as an important class of central nervous system depressants, acting as sedatives, anesthetics, and anticonvulsants [20]. However, it is known that they may also be of interest for pharmacology as antitumor [21] and anti-cancer [22] agents. Spirobarbiturates occupy a special place among barbituric acid derivatives, being not only valuable synthetic building blocks but also biologically important compounds. For example, spirobarbiturates show anticonvulsant and sedative effects [23,24] and also demonstrate activity as inhibitors of enzymes dihydro-orotate dehydrogenase [25] and collagenase-3 (MMP-13) [26]. Pre-clinical evidence suggests that inhibition of MMP-13 could be useful in osteoarthritis and rheumatoid arthritis [26]. It is worth noting here a promising representative of spirobarbiturates is Zoliflodacin (AZD0914, ETX0914), which has been shown to be a potential candidate for the treatment of urogenital gonorrhea [27] and *Mycoplasma genitalium* [28].

It was demonstrated in our recent papers that spiro-fused azabicyclo[3.1.0]hexanes and spiro-fused cyclopropa[*a*]pyrrolizines are easily accessible from azomethine ylides and cyclopropenes, and some of the cycloadducts suppressed the cancer cells grown in vitro [29,30,31,32,33]. It was also shown that they can affect cell motility and the cell cycle and induce apoptosis and morphological changes of cells; therefore, we assume that the screened compounds not only demonstrate antiproliferative activity, but also can lead to a decrease in tumor cells’ metastatic potential, which opens up broad prospects for the study of their action in vivo [34,35]. It is known from the literature that mechanisms of biological activity of compounds containing azabicyclo[3.1.0]hexane fragments may affect tumor protein p53 (indeed, TP53 gene is the most frequently mutated gene (>50%) in human cancer) and signal cascades such as STAT3/p-STAT3-JAK2/p-JAK2 as well as the target MDM2/p53 complex [36,37].

We report here the initial in vitro and in vivo evaluation of cycloadducts comprising spiro-fused barbituric acid and 3-azabicyclo[3.1.0]hexane frameworks, derived from one-pot three-component 1,3-dipolar cycloaddition reactions of in situ generated alloxan-derived azomethine ylides with various cyclopropenes.

## 2. Results and Discussion

### 2.1. Synthesis

**Scheme 1:**
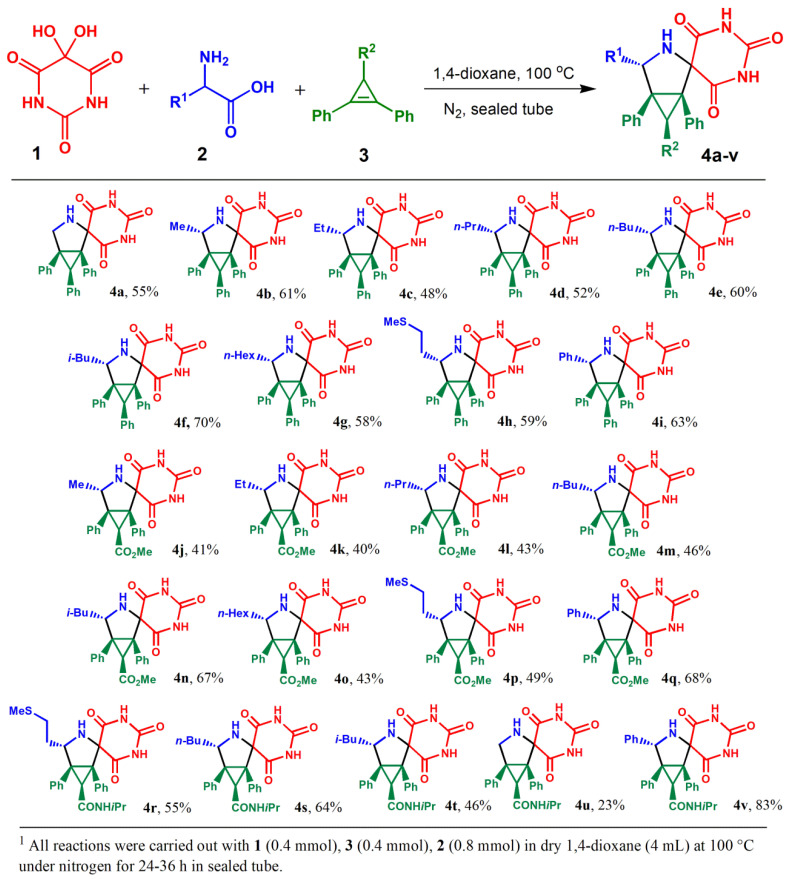
Synthesis of 3-azaspiro[bicyclo[3.1.0]hexane-2,5′-pyrimidines] **4** via one-pot three-component reactions of alloxane **1**, primary*α*-amino acids **2**, and cyclopropenes **3** ^1^.

Diastereoselective synthesis of biologically interesting complex alkaloid-like compounds with spiro-fused barbiturate and 3-azabicyclo[3.1.0]hexane moieties was described by us earlier and carried out according to our developed methodology via one-pot three-component 1,3-dipolar cycloaddition reactions of in situ generated alloxan-derived azomethine ylides with various cyclopropenes (Scheme 1). All the compounds were isolated by preparative thin-layer chromatography in yields up to 83% [38]. Additionally, for some of the most active compounds, the ADME parameters are given at Supplementary Materials (Tables S1 and S2).

### 2.2. Antiproliferative Effect of Synthesized Compounds against Cancer Cell Lines

**Figure 1:**
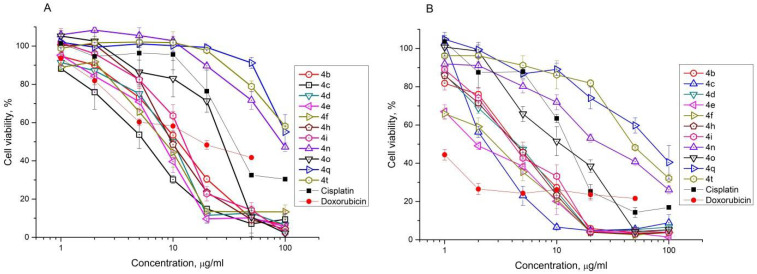
Cytotoxicity of selected 3-azaspiro[bicyclo[3.1.0]hexane-2,5′-pyrimidines] **4** against K562 cell line for 24 h (**A**) and 72 h (**B**).

**Figure 2:**
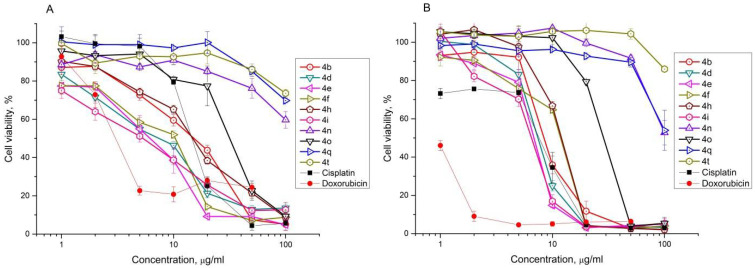
Cytotoxicity of selected 3-azaspiro[bicyclo[3.1.0]hexane-2,5′-pyrimidines] **4** against HeLa cell line for 24 h (**A**) and 72 h (**B**).

**Figure 3:**
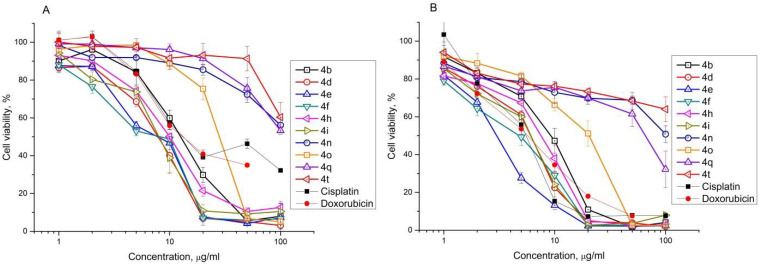
Cytotoxicity of selected 3-azaspiro[bicyclo[3.1.0]hexane-2,5′-pyrimidines] **4** against CT26 cell line for 24 h (**A**) and 72 h (**B**).

**Figure 4:**
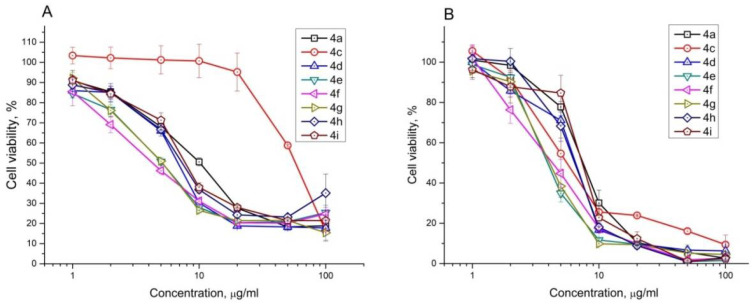
Cytotoxicity of 3-azaspiro[bicyclo[3.1.0]hexane-2,5′-pyrimidines] **4a,c**–**i** against Jurkat cell line for 24 h (**A**) and 72 h (**B**).

**Figure 5:**
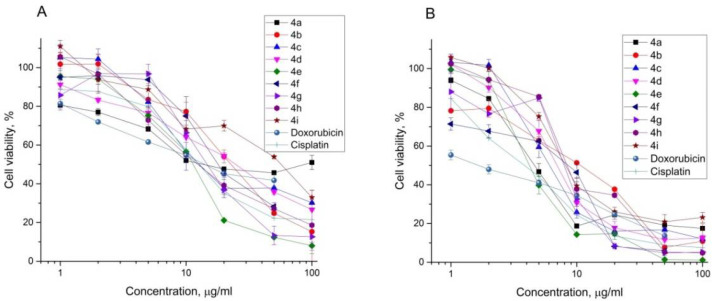
Cytotoxicity of 3-azaspiro[bicyclo[3.1.0]hexane-2,5′-pyrimidines] **4a**–**i** against Vero cell line for 24 h (**A**) and 72 h (**B**).

The cytotoxicity of synthesized 3-azaspiro[bicyclo[3.1.0]hexane-2,5′-pyrimidines] **4** against human erythroleukemia (K562), cervical carcinoma (HeLa), and T lymphocyte (Jurkat), as well as mouse colon carcinoma (CT26) and African green monkey kidney epithelial (Vero) cell lines as reference, was evaluated in vitro by the standard MTS assay for 24 and 72 h. The results are presented in Figure 1, Figure 2, Figure 3, Figure 4 and Figure 5 (data on all, tested on K562, HeLa, and CT26 cell lines’ compounds, are given at SI, Figures S1–S3). It was found that phenyl substituted at cyclopropane ring cycloadducts were more active in all cases (cf. **4a**–**i** vs. **4j**–**q** and **4r**–**v**). Replacement of the phenyl group by either the carboxymethyl or N-isopropylcarbamoyl group has led to a significant decrease in the activity of spiro-fused cycloadducts comprising azabicyclo[3.1.0]hexane and alloxane moieties. Thus, it was found that among target compounds with phenyl substituted at cyclopropane ring moiety **4b**–**i** (bearing methyl, ethyl, propyl, butyl, isobutyl, hexyl, 2-(methylthio)ethyl, and phenyl substituents, respectively), significant antiproliferative activity was demonstrated with IC_50_ ranging from 4 ± 2 to 14 ± 1, from 12 ± 6 to 70 ± 4, from 8 ± 2 to 19 ± 5, and from 3 ± 1 to 9 ± 1 μM after treatment for 72 h in K-562, HeLa, Jurkat, and CT26 cells, respectively (Table 1). As can be seen from the obtained results, the tested compounds have activity similar to such known compounds as Cisplatin and Doxorubicin.

Based on obtained data, the most active compounds were selected for further evaluation of the effects on cell motility, cytoskeletal morphology, cell cycle distribution, and cell death.

### 2.3. Cell Death Analysis

**Figure 6:**
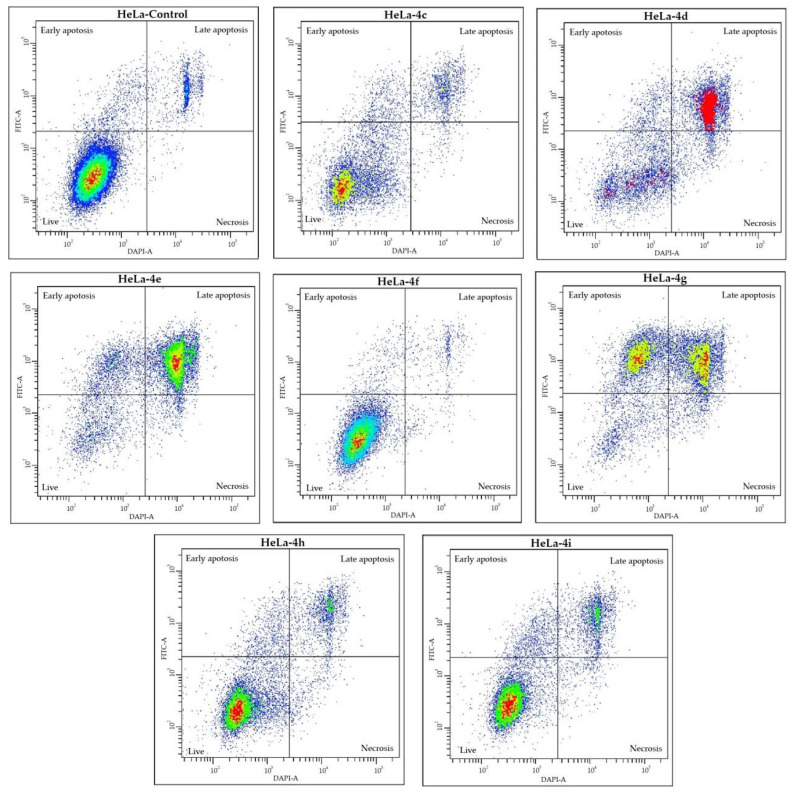
Apoptotic activity of HeLa cells treated with spiro-fused cycloadducts **4c–i** at concentration 10 μg/mL for 72 h using flow cytometry.

**Figure 7:**
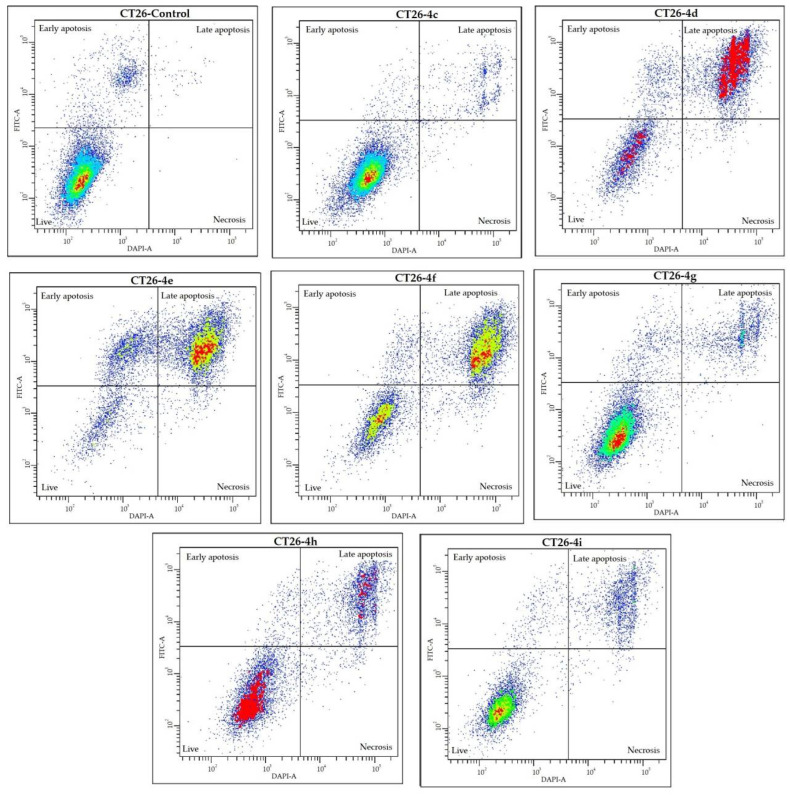
Apoptotic activity of CT26 cells treated with spiro-fused cycloadducts **4c–i** at concentration 10 μg/mL for 72 h using flow cytometry.

The results of cell death detection after the exposure of the HeLa and CT26 cell lines to spiro-fused cycloadducts **4c**–**i** at a dose of 10 μg/mL for 72 h, using Annexin V-FITC/DAPI staining, are shown in Figure 6 and Figure 7, respectively.

As shown in Figure 6 and Figure 7 and Table 2, the percentage of early and late apoptotic cells after treatment with compounds **4c**–**i** increased in all cases, except for **4c** and **4f** in HeLa cells and **4c** in CT26 cells. The percentage of early apoptotic cells increased from 4.5% (control) to 38.5% for HeLa cells and from 10.5% (control) to 23.4% for CT26 cells; the percentage of late apoptotic cells increased from 5.9% (control) to 38.1% and from 0.9% (control) to 61.6% for HeLa and CT26 cells, respectively.

The cycloadduct **4e** has the strongest effect in both cell lines: the percentage of live cells decreased nearly six times (from 89.0% and 88.5% to 15.0% and 13.5%, for HeLa and CT26, respectively). In addition, only compounds **4d** and **4g** caused significant cell death in the HeLa cell line (the percentage of live cells decreased nearly two and five times (from 89.0% to 46.8% and 17.1% for **4d** and **4g**, respectively)), while **4h** and **4i** showed just a minor effect (the percentage of live cells decreased from 89.0% to 72.2% and 67.9% for **4h** and **4i**, respectively). The rate of cell death in HeLa after exposure to compounds **4c** and **4f** was not different from the control.

Thus, it was indicated that compounds **4c**–**i** could induce apoptosis of HeLa and CT26 cells, and, at the same time, the compounds lead to inhibition of growth rates in these cells.

### 2.4. Cell Cycle Analysis

**Figure 8:**
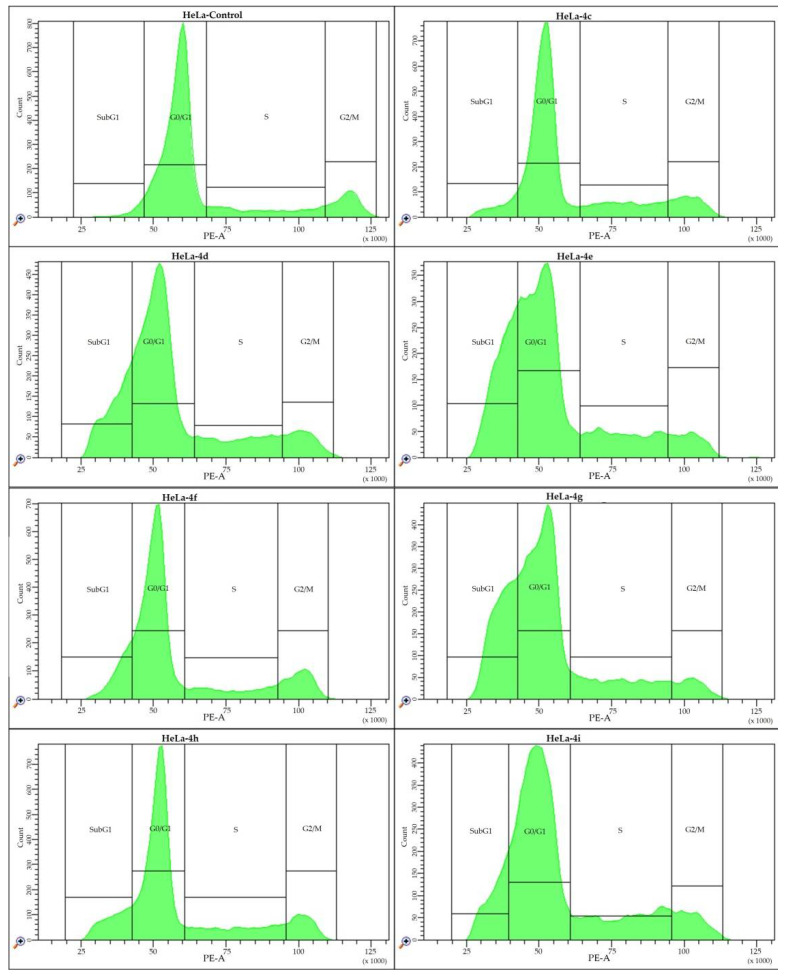
Effect of cycloadducts **4c**–**i** at concentration 10 μg/mL on the distribution of HeLa cells in the cell cycle.

**Figure 9:**
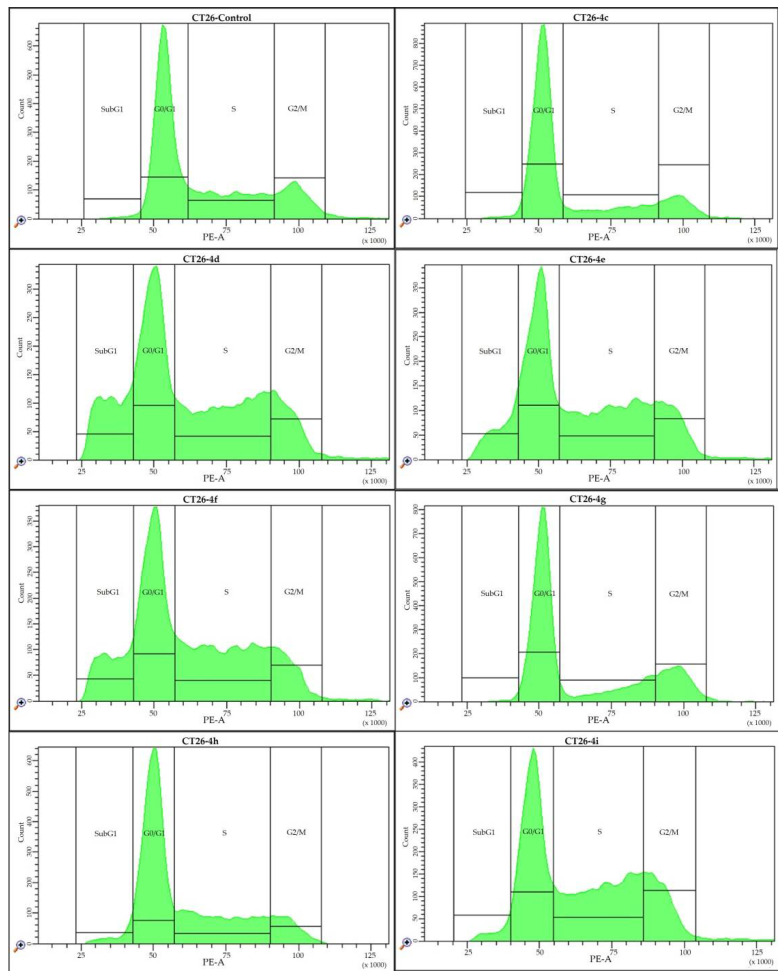
Effect of cycloadducts **4c**–**i** at concentration 10 μg/mL on the distribution of CT26 cells in the cell cycle.

**Figure 10:**
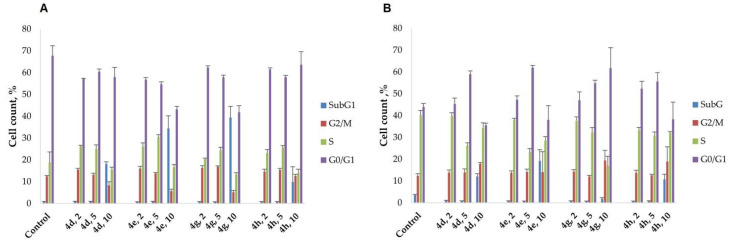
Effect of cycloadducts **4d**, **4e**, **4g**, and **4h** at concentrations 2, 5, and 10 μg/mL on the distribution of HeLa (**A**) and CT26 (**B**) cells in the cell cycle.

One of the indicators of the influence of biologically active products on the cells is the changed distribution of cells during different stages of the cell cycle. In this work, recently synthesized compounds were tested using flow cytometry for their effect on the distribution of cells in the cell cycle (G0/G1, G2/M, and S). Figure 8, Figure 9 and Figure 10 and Table 3 show the typical data after processing the results from three replicates of each experiment.

As it follows from the presented data, the number of HeLa cells in the S phase increased from 12.5% to 18.1%. The number of HeLa cells in the G2/M phase decreased from 12.2% to 5.3%. In CT26 cells, the number of cells in the S phase decreased from 40.1% to 17.0%, while the number of CT26 cells in the G2/M phase increased from 12.3% to 22.4%. In addition, cells treated with all the substances in **4** have a population in the SubG1 phase (up to 39.5 and 24.9% for HeLa and CT26 cells, respectively), which may indicate the activation of apoptosis. These data are in agreement with the results of cell death analysis. It should be noted that at 10 µg/mL, the substances displayed high cytotoxicity on tumor cells; however, at lower concentrations, they seem to inhibit cell cycle progression, as evidenced by the increased frequency of cells at the S- and G2/M-phases, respectively, in HeLa and CT26 cultures. The strongest cytostatic effect was observed after treatment with compounds **4e** and **4g** for the HeLa cell line, while **4d**, **4e**, **4f**, **4h**, and **4i** showed the best effect for the CT26 cell line.

### 2.5. Inhibition of Cell Motility Evaluated by Scratch Test

Cell motility is an ancient and basic cellular behavior that contributes to cancer invasion and metastasis [39]. An important task in metastatic-tumor-spread understanding is that the process cannot be straightly observed or manipulated. A scratch test is an easy model to evaluate the influence of different effects on cell motility and potential metastasis.

**Figure 11:**
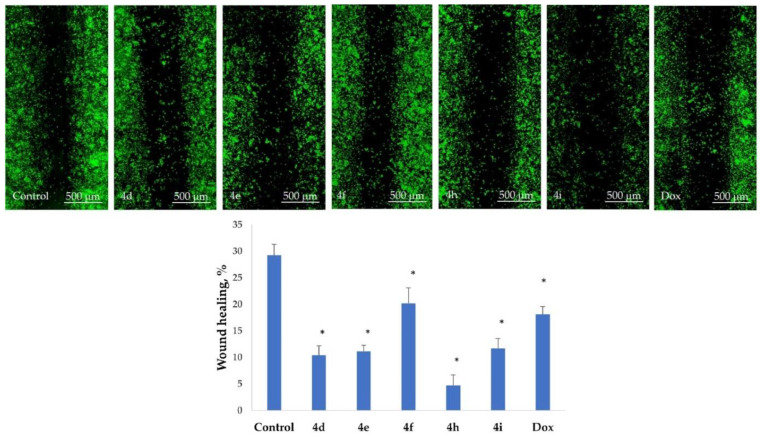
Microscopic images of the HeLa cells wound area in the scratch assay and wound area (%) in the scratch assay after 24 h incubation post azaspiro[bicycle[3.1.0]hexane-2,5′-pyrimidines] **4** treatment. Bars indicate ± SD; * *p* value < 0.05.

**Figure 12:**
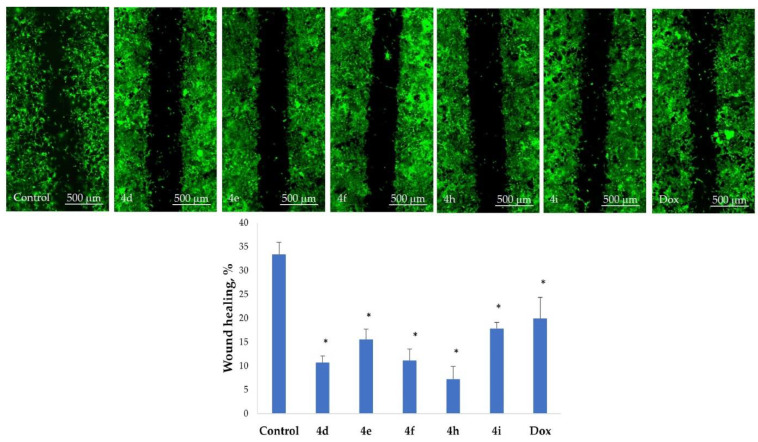
Microscopic images of the CT26 cells wound area in the scratch assay and wound area (%) in the scratch assay after 24 h incubation post azaspiro[bicycle[3.1.0]hexane-2,5′-pyrimidines] **4** treatment. Bars indicate ± SD; * *p* value < 0.05.

**Figure 13:**
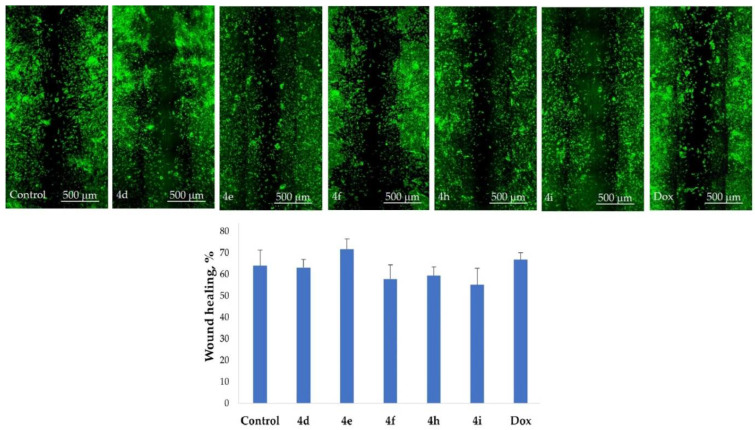
Microscopic images of the Vero cells wound area in the scratch assay and wound area (%) in the scratch assay after 24 h incubation post azaspiro[bicycle[3.1.0]hexane-2,5′-pyrimidines] **4** treatment. Bars indicate ± SD.

To evaluate the ability of tested adducts to inhibit cell-motility-associated metastasis, a scratch test was performed on the HeLa, CT26, and Vero cell lines. The results are shown in Figure 11, Figure 12 and Figure 13. Nontreated HeLa cells filled the scratched strip at 29.3 ± 2.0%, while cells filled 10.4 ± 1.8%, 11.2 ± 1.1%, 20.2 ± 2.9%, 4.8 ± 1.9%, and 11.7 ± 1.8% of the scratched strip under treatment with adducts **4d**, **4e**, **4f**, **4h**, and **4i**, respectively (Figure 11).

Nontreated CT26 cells filled the scratched strip at 33.4 ± 2.5%, while cells filled 10.7 ± 1.4%, 15.5 ± 2.2%, 11.1 ± 2.4%, and 7.2 ± 2.7% of the scratched strip under treatment with adducts **4d**, **4e**, **4f**, **4h**, and **4i**, respectively (Figure 12). The least inhibition effect was noticed for Vero cells, which was used as the control (Figure 13).

Therefore, both HeLa and CT26 cells lose their ability to move under treatment and do not fill the scratched strip. The presented results indicate that the tested compounds can block the cellular movement of tumor cells.

### 2.6. Actine Cytoskeleton Changes

It is known that the actin cytoskeleton has an essential role in vital cellular processes such as cell adhesion, migration, and morphogenesis [40,41]. Therefore, it may be used as an additional target for chemotherapeutic intervention [42,43]. Tumor transformation triggers reorganization of the actin cytoskeleton, which results in a change in cell motility. A correlation was observed between the increased migration activity of tumor cells and actin assembly and organization [44,45]. The structural features of actin organization can serve as the criteria for assessing the tumor cells metastatic potential [46]. The HeLa cell line is widely used to study the actin cytoskeleton structure. This cell line is characterized by the presence of actin stress fibers and filopodia [47,48].

**Figure 14:**
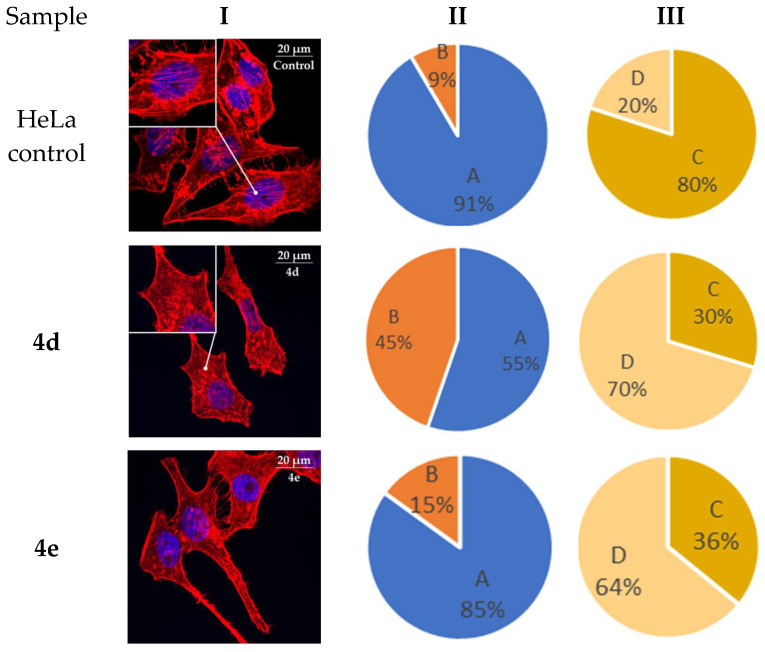

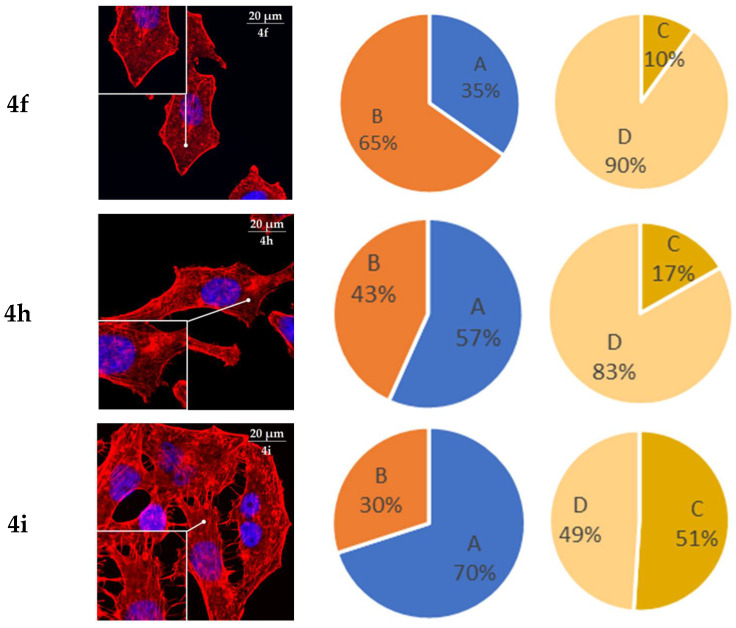
State of actin cytoskeleton of HeLa cells after treatment with 3-azaspiro[bicyclo[3.1.0]hexane-2,5′-pyrimidines] **4d**, **4e**, **4f**, **4h**, and **4i**. I: Images demonstrate the different stages of cell actin cytoskeleton. II: Pie charts demonstrate percentage of cells with filopodia-like deformations (A) and without filopodia-like deformations (B). III: Pie charts demonstrate percentage of cells with normal stress fibers (C) and disassembled stress fibers (D).

**Figure 15:**
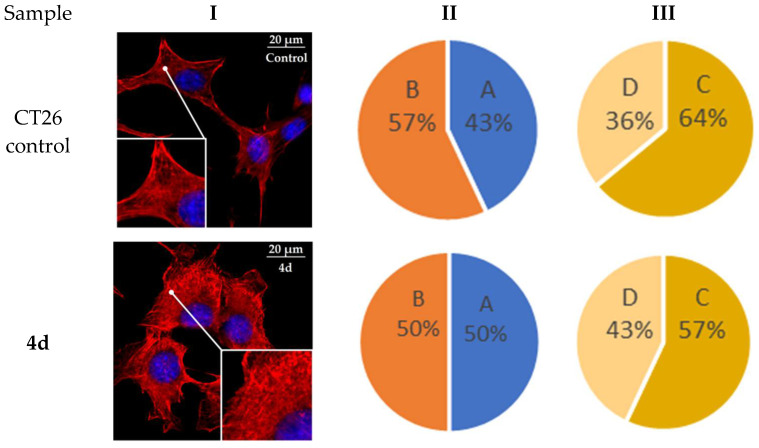

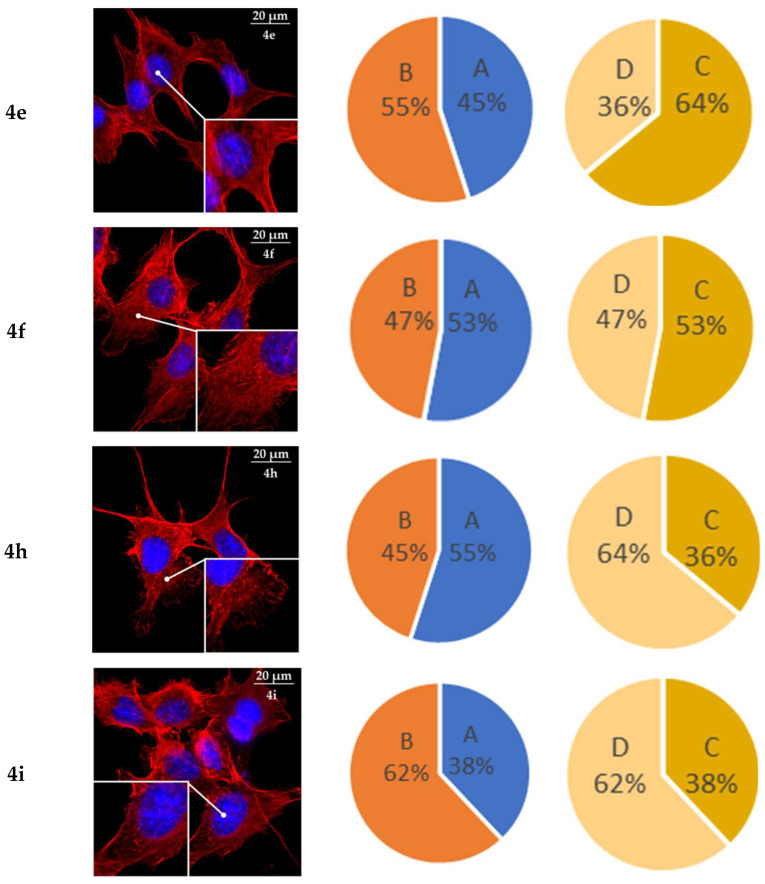
State of actin cytoskeleton of CT26 cells after treatment with 3-azaspiro[bicyclo[3.1.0]hexane-2,5′-pyrimidines] **4d**, **4e**, **4f**, **4h**, and **4i**. I: Images demonstrate the different stages of cell actin cytoskeleton. II: Pie charts demonstrate percentage of cells with filopodia-like deformations (A) and without filopodia-like deformations (B). III: Pie charts demonstrate percentage of cells with normal stress fibers (C) and disassembled stress fibers (D).

**Figure 16:**
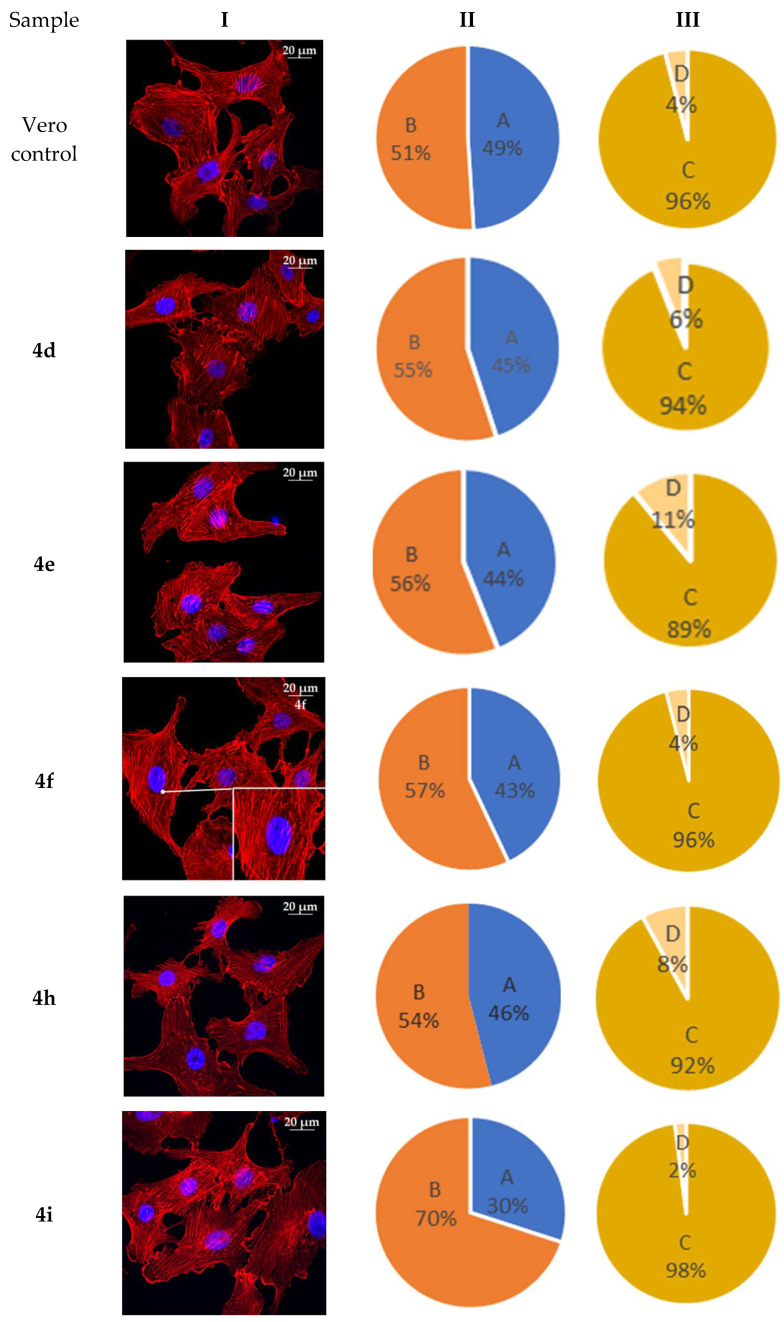
State of actin cytoskeleton of Vero cells after treatment with 3-azaspiro[bicyclo[3.1.0]hexane-2,5′-pyrimidines] **4d**, **4e**, **4f**, **4h**, and **4i**. I: Images demonstrate the different stages of cell actin cytoskeleton. II: Pie charts demonstrate percentage of cells with filopodia-like deformations (A) and without filopodia-like deformations (B). III: Pie charts demonstrate percentage of cells with normal stress fibers (C) and disassembled stress fibers (D).

Therefore, in the current study, the actin cytoskeleton structure of HeLa, CT26, and Vero cells was analyzed, after the impact of compounds **4a**–**i**, by the presence of filopodia-like protrusions and the availability of stress fibers (Figure 14, Figure 15 and Figure 16). It was found that treatment with cycloadducts **4** has led to a significant alteration in the tumor cells’ actin cytoskeleton structure, which leads to the changes in the number of filopodia-like deformations and stress fibers’ disappearance. HeLa cells were more sensitive to the action of tested products, compared to CT26 and Vero cells. Such treatment of HeLa cells with compounds **4d, 4e, 4f, 4h**, and **4i** resulted in the decrease in the number of cells with stress fibers from 80% down to 10% (for product 4f), while, for CT26 cells, the number of cells with stress fibers decreased from 64% to 36% and 38% (for products **4h** and **4i**, respectively). Similarly, the number of cells with filopodia-like structures decreased to 35% in HeLa cells treated with the most potent adduct **4f** (as compared to 91% in the control sample) and to 38% in CT26 cells (for the most potent adduct **4i**, compared to 43% in the control sample).

### 2.7. In Vivo Evaluation

**Figure 17:**
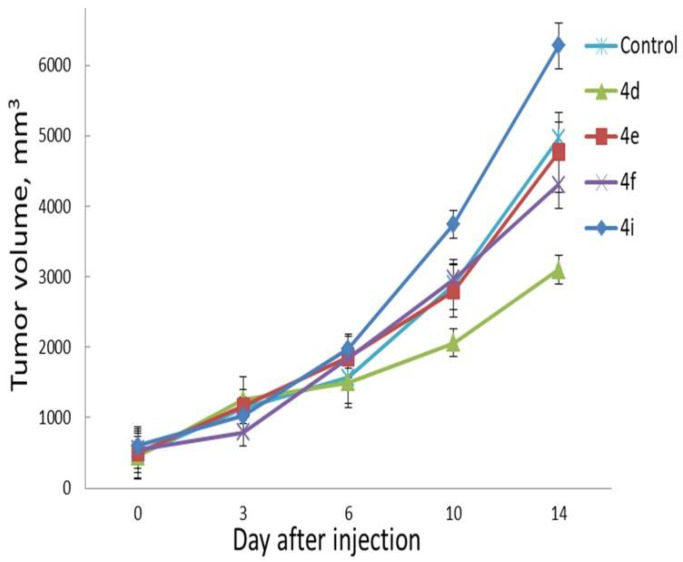
Dynamics of tumor growth with a single intraperitoneal administration of drugs based on 3-azaspiro[bicyclo[3.1.0]hexane-2,5′-pyrimidines] **4d**, **4e**, **4f**, and **4i**. Bars indicate ±SD.

In vivo studies were performed using CT26 tumor-bearing Balb/C mice. After the average tumor volume reached approximately 270 mm^3^, the mice were injected intraperitoneally with 300 μL of 3 μM suspension of compounds **4d, 4e, 4f**, and **4i** in 10% aq. DMSO solution. Mice in the control group were injected with 300 μL of 10% aq. DMSO solution. Observation of the animals continued for 10 days, with the measurement of tumor sizes two times a week. Results are presented in Figure 17.

At the 10th day of the experiment, no statistically significant differences were observed between the corresponding control and experimental groups of animals. At the same time, by the end of the observation period, the death of experimental animals in the control group (one animal) and the **4e** (two animals), **4f** (one animal), and **4i** (three animals) groups was noted. No deaths were registered in group **4d** during the entire observation period. In the surviving animals, there was no appetite suppression, behavioral reaction, weight loss, excretory dysfunction, or pathological change in the coat or at the injection site.

By the end of the experiment, all animals were euthanized, with a subsequent autopsy analysis of the state of the abdominal organs. At the same time, macroscopic signs of acute toxicity were not detected: there was no adhesive process, sign of focal peritonitis, hemorrhagic change in the peritoneum and mucous membranes of hollow organs, area of aseptic inflammation, or swelling of parenchymal organs (liver and kidneys). All animals showed sufficiently developed fatty tissue of the omentum, in the paravertebral region, and in the perinephric compartment.

**Figure 18:**
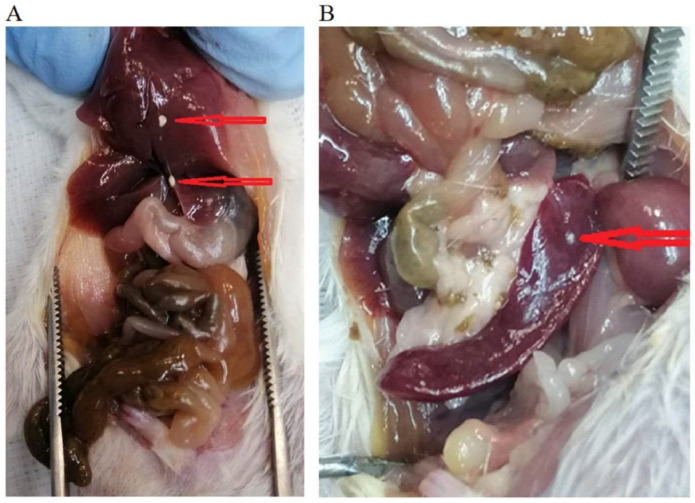
Organs of the mouse abdominal cavity during autopsy. Animal #12 (group 1, **4e**). Macropreparation. There are no signs of local inflammation, adhesion, or sufficient development of adipose tissue. (**A**)—x 2. No signs of acute intoxication. The arrows indicate the agglomerates of the injected compound observed on the surface of the liver. (**B**)—x 4. The area of the gastrointestinal angle and the left kidney. The arrows indicate the agglomerates of the injected compound observed on the surface of the spleen.

At the same time, in animals receiving intraperitoneal injection of solutions of compounds **4d**, **4e**, and **4i**, the appearance of persistent, rounded, dense agglomerates of white or yellowish-white foreign material, with a diameter of up to 1 mm, fixed under the peritoneum to varying degrees of severity, was noted (Figure 18).

The typical location for the localization of agglomerates of the injected compounds was the sheets of the peritoneum of the omentum and the pancreas, followed by the spleen, liver capsule, and pelvic organs. By mass spectrometric analysis, it was found that these inclusions were represented by the material of the injected compounds that precipitated, likely due to rapid absorption of the solvent used (DMSO), from the injection site.

Thus, the antitumor effect of the studied compounds on the model of the transplanted CT26 tumor at the selected concentration and single administration mode could not be detected. At the same time, low mortality and the absence of signs of systemic and local toxicity noted during parenteral administration allow for the continuation of the study of the compounds containing spiro-fused barbiturate and 3-azabicyclo-[3.1.0]hexane moieties.

## 3. Materials and Methods

### 3.1. Cell Culture and Culturing Conditions

All the cell lines (human cervical carcinoma (HeLa), erythroleukemia (K-562), and T lymphocyte (Jurkat) as well as mouse colon carcinoma (CT26) and African green monkey kidney epithelial (Vero)) were obtained from the cell repository “Vertebrate cell culture collection” (supported by the Ministry of Science and Higher Education of the Russian Federation, agreement №075-15-2021-683, Institute of Cytology, Russian Academy of Sciences, Saint Petersburg, Russia). HeLa, Jurkat, and Vero cells were cultured in DMEM (HyClone, South Logan, UT, USA) supplemented with 10% (*v*/*v*) fetal bovine serum (Hyclone, GE Healthcare Life Sciences, Logan, UT, USA), and gentamicin (Sigma-Aldrich, St. Louis, MO, USA) at 37 °C in a humidified atmosphere with 5% CO_2_. K-562 and CT26 cells were grown at RPMI medium (Hyclone, GE Healthcare Life Sciences, Logan, UT, USA), with the same supplements and conditions.

### 3.2. Cell Proliferation Assay

To evaluate the in vitro toxicity of compounds synthesized, cells were seeded into 96-well plates at a density of 5 × 10^3^ cells per well. On the next day, tested compounds were added to the wells at concentrations ranging from 1 to 100 mg/mL, followed by incubation for 1 and 3 days. Cell proliferation was determined by adding 20 μL of MTS reagent (BioVision, Milpitas, CA, USA) stock solution per well. Each plate was incubated for 2 h at 37 °C in a humidified, 5% CO_2_ atmosphere. The plates were then read at 495 nm using plate spectrophotometer (Multiskan GO, Thermo Fisher Scientific, Waltham, MA, USA). All samples were measured in triplicates.

### 3.3. Cell Distribution over the Different Phases of the Cell Cycle

The distribution of HeLa and CT26 cells in the G0/G1-, S-, and G2/M-phases of the cell cycle was obtained by quantification of DNA content in propidium-iodide-stained cells using flow cytometry. Briefly, cells were seeded in 24-well plates at a density of 5 × 10^4^ cells per well. After 24 h incubation, cells were exposed to 10 μg/mL of compounds **4c**–**i** (the structure is provided in Scheme 1) for 24 h. The effects of compounds **4d**, **4e**, **4g**, **4h** were additionally tested at concentrations of 2 and 5 μg/mL. After incubation with drugs, the cells were detached by exposure to trypsin-EDTA for 5 min at 37 °C and then collected by pipetting. This was followed by treatment with 0.2 mg/mL saponin (Fluka, Waltham, MA, USA), 0.25 mg/mL RNase (Sigma-Aldrich, St. Louis, MO, USA), and 0.05 mg/mL propidium iodide (Invitrogen, Carlsbad, CA, USA). After washing, the samples were analyzed by standard flow cytometer (BD FACSCanto II, Becton Dickinson, San Jose, CA, USA). Then, 10,000 events were acquired for the sample. Data processing was performed using BD FACSDiva 9.0 software.

### 3.4. Annexin V-FITC/DAPI Staining Assay

Cells were seeded in 24-well plates, 5 × 10^4^ cells per well. After 24 h incubation, cells were treated with compounds **4c**–**i** for 72 h. Then, cells were washed, harvested by trypsinisation, stained with Annexin V-FITC (BD FACSCanto II, Becton Dickinson, San Jose, CA, USA) and DAPI (Thermo Fisher Scientific, Waltham, MA, USA), in accordance with the protocol of the manufacturer, and analyzed by flow cytometry. The proapoptotic effect of azaspiro[bicyclo[3.1.0]hexane-2,5′-pyrimidines] **4c**–**i** was evaluated by an Annexin V-FITC/DAPI (AV/DAPI) dual-staining assay to examine the occurrence of phosphatidylserine externalization, which facilitated the detection of live cells (lower-left quadrant; AV−/DAPI−), early apoptotic cells (upper-left quadrant; AV+/DAPI−), late apoptotic cells (upper-right quadrant; AV+/DAPI+), and necrotic cells (lower-right quadrant; AV−/DAPI+) [49].

### 3.5. Actin Cytoskeleton Staining

Cells were seeded onto Petri dishes with cover slips at a density of 2 × 10^5^ cells per dish and incubated for 24 h. After that, cells were treated with compounds **4d**, **4e**, **4f**, **4h**, and **4i** (5 μg/mL) for 24 h. The medium was removed, and cells were fixed with 4% paraformaldehyde (Sigma-Aldrich, St. Louis, MO, USA), washed three times with PBS, and permeabilized with 0.3% Triton-X100 (Sigma-Aldrich, St. Louis, MO, USA). The cells were rinsed three times with PBS. Actin filaments (microfilaments) were stained at 37 °C for 15 min with rhodamine-phalloidin (Invitrogen, Carlsbad, CA, USA). The samples were rinsed three times with PBS, followed by embedding in Fluoroshield medium (Sigma-Aldrich, St. Louis, MO, USA). Cells were imaged using an Axio Observer Z1 confocal microscope (Carl Zeiss MicroImaging GmbH, Jena, Germany). In each experiment, at least 30 cells were imaged. Images were analyzed by a pathologist, blinded to the treatment mode used for each group, using ImageJ software.

### 3.6. Evaluation of Cell Motility by Scratch Test

Cells were seeded onto Petri dishes at a density of 5 × 10^5^ cells per dish and grown to confluency. Scratch wounds were made with a 200 μL pipette tip, after which detached cells were removed by washing with phosphate-buffered saline. In order to inhibit cell proliferation, culture media was replaced to serum-free DMEM. Compounds **4d**, **4e**, **4f**, and **4h** were added to the cultures at a dose of 10 μg/mL and incubated for 24 h. After that, the cells were stained with Hoechst 33,342 (Thermo Fisher Scientific, Waltham, MA, USA), by adding 2 μL of 1 mg/mL stock solution to 2 mL of medium and DIBAC4 (3) (Thermo Fisher Scientific, Waltham, MA, USA) at the same dose. Images were captured using confocal microscope (Axio Observer Z1, Carl Zeiss MicroImaging GmbH, Jena, Germany). The percentage of wound closure in five randomly chosen fields was calculated with NIH ImageJ software.

### 3.7. Laboratory Animals and Ethics Statement

All animals were bred and maintained in specific pathogen-free facilities in accordance with the Rus-LASA and FELASA guidelines. This study complied with all relevant ethical regulations for animal testing and research and received ethical approval from the Almazov National Medical Research Centre (protocol No. 21-02, dated 26 March 2021). Animal experiments were carried out in accordance with the principles of humane treatment of animals, regulated by requirements of Council of Europe 2006; European Convention for the Protection of Vertebrate Animals Used for Experimental and other Scientific Purposes (ETS No. 123); the European Parliament and the Council of the European Union 2010; Directive 2010/63/EU of the European Parliament and of the Council of 22 September 2010 on the Protection of Animals Used for Scientific Purposes; and FELASA guidelines. Male, 20–22 g, SPF Balb/C mice (Pushchino, Moscow region, Russian Federation) were used throughout. Animals were maintained at 22 ± 2 °C and relative humidity 50 ± 10% with 12 h light/dark cycle. All mice received water and food ad libitum. Animals were checked daily by the veterinarian, and their state of health was monitored continuously. Animal body weight was recorded every three days (GX-600 Precision Balance, A&D Weighing, San Jose, CA, USA).

### 3.8. Assessment of Anti-Tumor Effect In Vivo

The greatest transverse diameter (width) and the greatest longitudinal diameter (length) of a tumor were determined with a Vernier mechanical caliper. The tumor volume (V) was calculated using the following formula: V = length × width 2/2000 [50]. Mice were inoculated subcutaneously in the right flank, with 2 × 10^6^ CT26 cells per mouse in PBS (Corning, Glendale, AZ, USA). Ten days later, when the average tumor volume reached 0.52 ± 0.15 cm^3^, the mice were randomized into groups (*n* = 7 per group). An amount of 300 μL of 3 μM suspension of compounds **4d**, **4e**, **4f**, and **4i** in 10% aq. DMSO solution was injected intraperitoneally at once. The control group was injected with 10% aq. DMSO solution only. The tumor volumes were monitored as described above, along with body weight. Mice were euthanized when the subcutaneous tumor reached a volume of ~10 cm^3^.

### 3.9. Statistical Analysis

Statistical processing of results was performed using Statistica 6.0. All data from the three independent experiments were used for measuring the means ± standard deviation (mean ± SD) that were compared using the Student’s *t*-test or nonparametric Wilcoxon Mann–Whitney *U* test. Differences among groups were considered significant at *p* ≤0.05.

## 4. Conclusions

We have studied a series of spiro-fused heterocyclic compounds containing barbiturate and 3-azabicyclo[3.1.0]hexane moieties as potential antitumor agents. The antiproliferative activity of the products was screened against human erythroleukemia (K562), T lymphocyte (Jurkat), and cervical carcinoma (HeLa) as well as mouse colon carcinoma (CT26) and African green monkey kidney epithelial (Vero) cell lines. Most effective among the screened compounds showed IC_50_ in the range from 4.2 to 24.1 μM for all tested cell lines. Replacement of the phenyl group of cyclopropane moiety with either the alkoxycarbonyl or diisopropylcarbamoyl groups leads to a significant decrease in the activity of the formed cycloadducts (compare **4j**–**q** and **4r**–**v** vs. **4a**–**i**). The substituent at the pyrrolidine moiety has less impact on the activity. The usually unsubstituted adduct **4a** is less active as compared to other phenyl substituted at cyclopropane moiety adducts **4b**–**i**. At the same time, there is not a significant difference in cell viability under treatment with cycloadducts that bear alkyl or thioalkyl or aryl (phenyl) substituent at pyrrolidine moiety (**4b**–**i**). In agreement with the DNA cytometry studies, the screened compounds have demonstrated the significant effect of the distribution of HeLa and CT26 cells across cell cycle stages with an accumulation of cells in the SubG1 phase and also of cells entering apoptosis. It was found, using confocal microscopy, that actin filaments disappeared and granular actin was distributed diffusely in the cytoplasm of up to 90% of HeLa cells and up to 64% of CT26 cells, after their treatment with tested 3-azaspiro[bicyclo[3.1.0]hexane-2,5′-pyrimidines]. We showed that the number of HeLa cells with filopodium-like membrane protrusions was reduced significantly (from 91% in control cells to 35%), after treatment with the most active compounds. Furthermore, a decrease in cell motility was observed. Preliminary in vivo experiments on the dynamics of CT26 tumor growth in Balb/C mice showed no statistically significant differences between the corresponding control and experimental groups of animals, after single intraperitoneal administration of cycloadducts. At the same time, low mortality and the absence of signs of systemic and local toxicity noted during parenteral administration allows to continue the anti-tumor effects of the compounds containing spiro-fused barbiturate and 3-azabicyclo-[3.1.0]hexane moieties.

## Figures and Tables

**Table 1:** IC_50_ value for 3-azaspiro[bicyclo[3.1.0]hexane-2,5′-pyrimidines] **4b**–**i**.

Compound	IC_50_, μM													
	K562			HeLa			CT26			Jurkat			Vero	
	24 h	72 h	SI	24 h	72 h	SI	24 h	72 h	SI	24 h	72 h	SI	24 h	72 h
**4a**	47 ± 8	14 ± 1	1.0	125 ± 18	70 ± 4	0.2	64 ± 7	50 ± 7	0.3	24 ± 2	19 ± 5	0.8	50 ± 14	14 ± 2
**4b**	25 ± 5	9 ± 2	2.4	34 ± 2	18 ± 2	1.2	30 ± 2	21 ± 2	1.0	-	-	-	55 ± 7	21 ± 2
**4c**	12 ± 2	5 ± 2	3.2	21 ± 2	13 ± 2	1.3	27 ± 2	20 ± 2	0.8	149 ± 33	15 ± 2	1.1	47 ± 11	16 ± 2
**4d**	21 ± 4	9 ± 2	1.8	17 ± 2	15 ± 2	1.1	17 ± 2	13 ± 2	1.3	15 ± 2	13 ± 2	1.3	48 ± 4	16 ± 2
**4e**	17 ± 2	4 ± 2	2.5	13 ± 2	13 ± 4	0.8	10 ± 2	6 ± 2	1.7	12 ± 2	9 ± 2	1.2	23 ± 2	10 ± 2
**4f**	17 ± 4	6 ± 2	1.7	19 ± 2	23 ± 6	0.5	17 ± 2	10 ± 2	1.0	11 ± 2	9 ± 2	1.2	48 ± 6	10 ± 2
**4g**	19 ± 2	10 ± 2	1.4	15 ± 2	13 ± 2	1.1	22 ± 2	12 ± 2	1.2	11 ± 2	8 ± 2	1.8	33 ± 6	14 ± 2
**4h**	20 ± 4	10 ± 2	2.2	28 ± 2	24 ± 6	1.0	22 ± 2	16 ± 2	1.4	18 ± 4	13 ± 4	1.7	31 ± 4	22 ± 4
**4i**	26 ± 4	8 ± 4	2.9	10 ± 2	12 ± 6	2.0	16 ± 2	12 ± 2	2.0	19 ± 2	14 ± 4	1.7	97 ± 12	23 ± 4
**Cisplatin**	46 ± 8	14 ± 2	0.3	16 ± 4	6 ± 1	0.7	24 ± 4	5 ± 1	0.8	-	-	-	14 ± 1	4 ± 1
**Doxorubicin**	16 ± 3	1.2 ± 0.3	2.0	4 ± 1	0.5 ± 0.1	4.8	19 ± 3	5.5 ± 0.3	0.5	-	-	-	13 ± 2	2.4 ± 0.4

SI—selectivity index.

**Table 2:** Apoptotic activity of HeLa (A) and CT26 (B) cells under the treatment with 3-azaspiro[bicyclo[3.1.0]hexane-2,5′-pyrimidines] **4c**–**i**.

A	**HeLa**	**Live Cells, %**	**Early Apoptotic Cells, %**	**Late Apoptotic Cells, %**	**Necrotic Cells, %**
	Control	89.0 ± 0.7	4.5 ± 0.7	5.9 ± 0.4	0.5 ± 0.3
	**4c**	81.0 ± 4.4 *	5.6 ± 1.3	1.9 ± 2.8 *	2.2 ± 0.5 *
	**4d**	46.8 ± 18.4 *	11.6 ± 0.2 *	38.1 ± 16.8 *	3.6 ± 1.8 *
	**4e**	15.0 ± 2.7 *	18.1 ± 3.1 *	61.3 ± 3.8 *	5.6 ± 2.6 *
	**4f**	90.9 ± 1.6	2.9 ± 0.1 *	4.1 ± 0.5 *	2.1 ± 1.3 *
	**4g**	17.1 ± 3.5 *	38.5 ± 1.9 *	38.0 ± 6.9 *	6.3 ± 2.8 *
	**4h**	72.2 ± 0.4 *	9.8 ± 1.0 *	15.3 ± 0.5 *	1.9 ± 0.2 *
	**4i**	67.9 ± 2.4 *	12.6 ± 0.8 *	17.3 ± 1.4 *	2.1 ± 0.9 *
B	**CT26**	Live Cells, %	Early Apoptotic Cells, %	Late Apoptotic Cells, %	Necrotic Cells, %
	Control	88.5 ± 1.1	10.5 ± 0.7	0.9 ± 0.4	0.0 ± 0.0
	**4c**	89.8 ± 0.5	1.9 ± 0.3 *	6.4 ± 1.0 *	1.8 ± 0.8 *
	**4d**	32.8 ± 3.0 *	9.6 ± 0.5	56.4 ± 2.4 *	1.1 ± 0.3 *
	**4e**	13.5 ± 0.4 *	23.4 ± 0.8 *	61.6 ± 0.5 *	1.4 ± 0.1 *
	**4f**	34.1 ± 3.8 *	6.2 ± 1.2 *	57.8 ± 3.1 *	1.9 ± 0.5 *
	**4g**	84.2 ± 0.3 *	5.5 ± 0.1 *	10.0 ± 0.3 *	0.3 ± 0.1 *
	**4h**	70.3 ± 0.6 *	4.8 ± 0.5 *	24.0 ± 0.7 *	0.9 ± 0.3 *
	**4i**	76.2 ± 0.3 *	4.0 ± 0.1 *	19.5 ± 0.5 *	0.4 ± 0.1 *

Statistical differences between treated and control groups indicated by *.

**Table 3:** Effect of cycloadducts **4c**–**i** at a concentration of 10 μg/mL on the distribution of HeLa (A) and CT26 (B) cells during the cell cycle.

A	**HeLa**	**Control**	**4c**	**4d**	**4e**	**4f**	**4g**	**4h**	**4i**
	G0/G1 (%)	73.1 ± 2.6	63.3 ± 2.2 *	58.0 ± 4.4 *	43.1 ± 1.4 *	61.5 ± 3.3 *	41.8 ± 3.0 *	63.6 ± 6.8 *	51.8 ± 4.0 *
	S (%)	12.5 ± 1.7	17.6 ± 1.2 *	15.5 ± 1.1 *	16.7 ± 1.2 *	15.2 ± 1.2 *	13.3 ± 0.8	13.9 ± 1.8	18.1 ± 2.4 *
	G2/M (%)	12.2 ± 1.3	11.0 ± 1.5	8.4 ± 1.5 *	5.7 ± 0.9 *	12.8 ± 1.3	5.3 ± 0.7 *	12.6 ± 0.8	9.9 ± 0.8 *
	SubG1 (%)	2.3 ± 0.4	8.1 ± 1.9 *	18.1 ± 1.0 *	34.4 ± 5.8 *	10.5 ± 2.0 *	39.5 ± 5.0 *	9.9 ± 7.8 *	20.1 ± 1.3 *
B	CT26	Control	**4c**	**4d**	**4e**	**4f**	**4g**	**4h**	**4i**
	G0/G1 (%)	44.0 ± 1.7	68.5 ± 3.0 *	35.6 ± 1.0 *	38.1 ± 6.4 *	29.7 ± 1.4 *	61.7 ± 9.4 *	38.3 ± 7.9 *	33.2 ± 0.6 *
	S (%)	40.1 ± 1.5	16.3 ± 1.7 *	34.4 ± 2.3 *	28.5 ± 1.8 *	31.9 ± 2.6 *	17.0 ± 4.3 *	32.0 ± 0.6 *	37.2 ± 0.2 *
	G2/M (%)	12.3 ± 0.9	13.2 ± 2.9 *	17.9 ± 0.6 *	14.1 ± 9.4	13.5 ± 0.9 *	19.4 ± 4.7 *	18.9 ± 6.8 *	22.4 ± 1.7 *
	SubG1 (%)	3.6 ± 0.6	2.0 ± 0.4 *	12.2 ± 1.1 *	19.3 ± 5.1 *	24.9 ± 5.2 *	1.9 ± 0.5 *	10.8 ± 2.3 *	7.2 ± 2.2 *

Statistical differences between treated and control groups indicated by *.

## Data Availability

The data presented in this study are available on request from the corresponding authors.

## Supplementary Materials

The following supporting information can be downloaded at: https://www.mdpi.com/article/10.3390/ijms231810759/s1.
