# Supplementary material for: Biological Evaluation of 3-Azaspiro[Bicyclo[3.1.0]Hexane-2,5′-Pyrimidines] as Potential Antitumor Agents
Source: Int J Mol Sci. 2022 Sep 15;23(18):10759. doi: 10.3390/ijms231810759 (PMC9506420; doi:10.3390/ijms231810759)
Supplement: Supplementary file 1 [file ijms-23-10759-s001.zip › ijms-1885220-supplementary.pdf]

Supporting information

Table of contents

1. Cytotoxicity of 3-azaspiro[bicyclo[3.1.0]hexane-2,5'-pyrimidines] **4a-v** against K562, HeLa and CT26 cell lines. **S2**

2. In Silico Study **S5**

1. Cytotoxicity of 3-azaspiro[bicyclo[3.1.0]hexane-2,5'-pyrimidines] 4a-v against K562, HeLa and CT26 cell lines

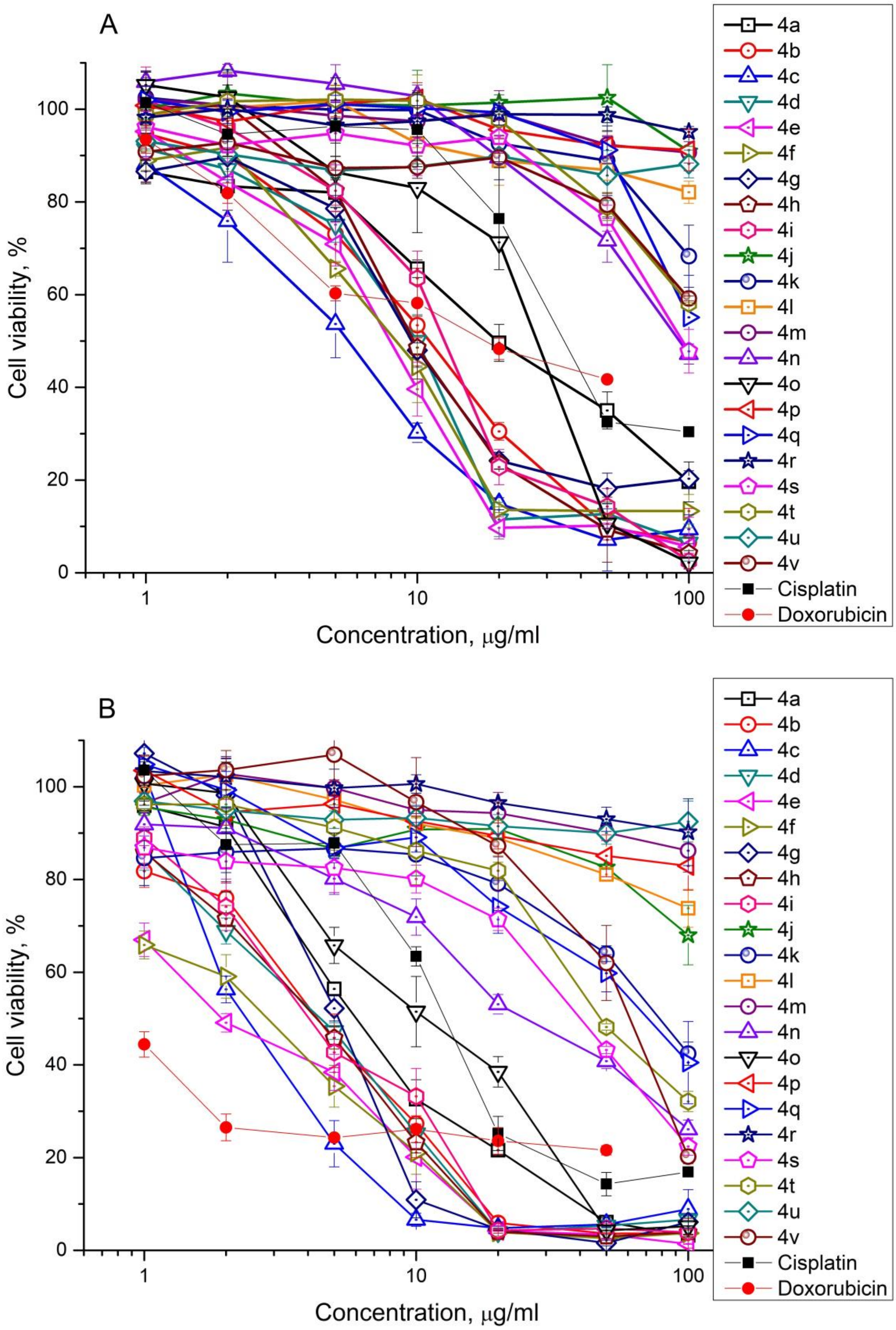

Figure S1. Cytotoxicity of 3-azaspiro[bicyclo[3.1.0]hexane-2,5′-pyrimidines] **4a-v** against K562 cell line for 24 h (A) and 72 h (B).

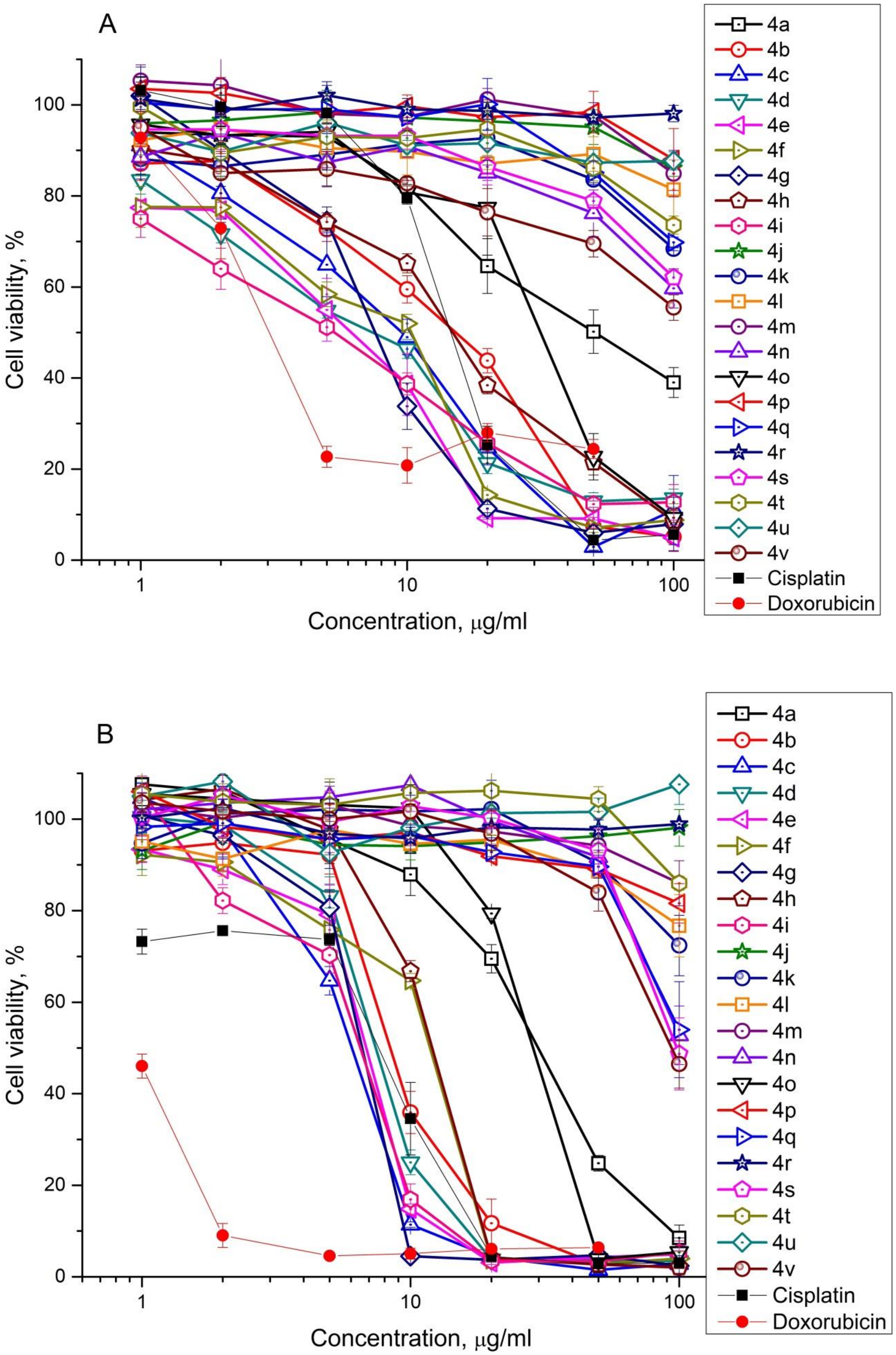

Figure S2. Cytotoxicity of 3-azaspiro[bicyclo[3.1.0]hexane-2,5′-pyrimidines] **4a-v** against HeLa cell line for 24 h (A) and 72 h (B).

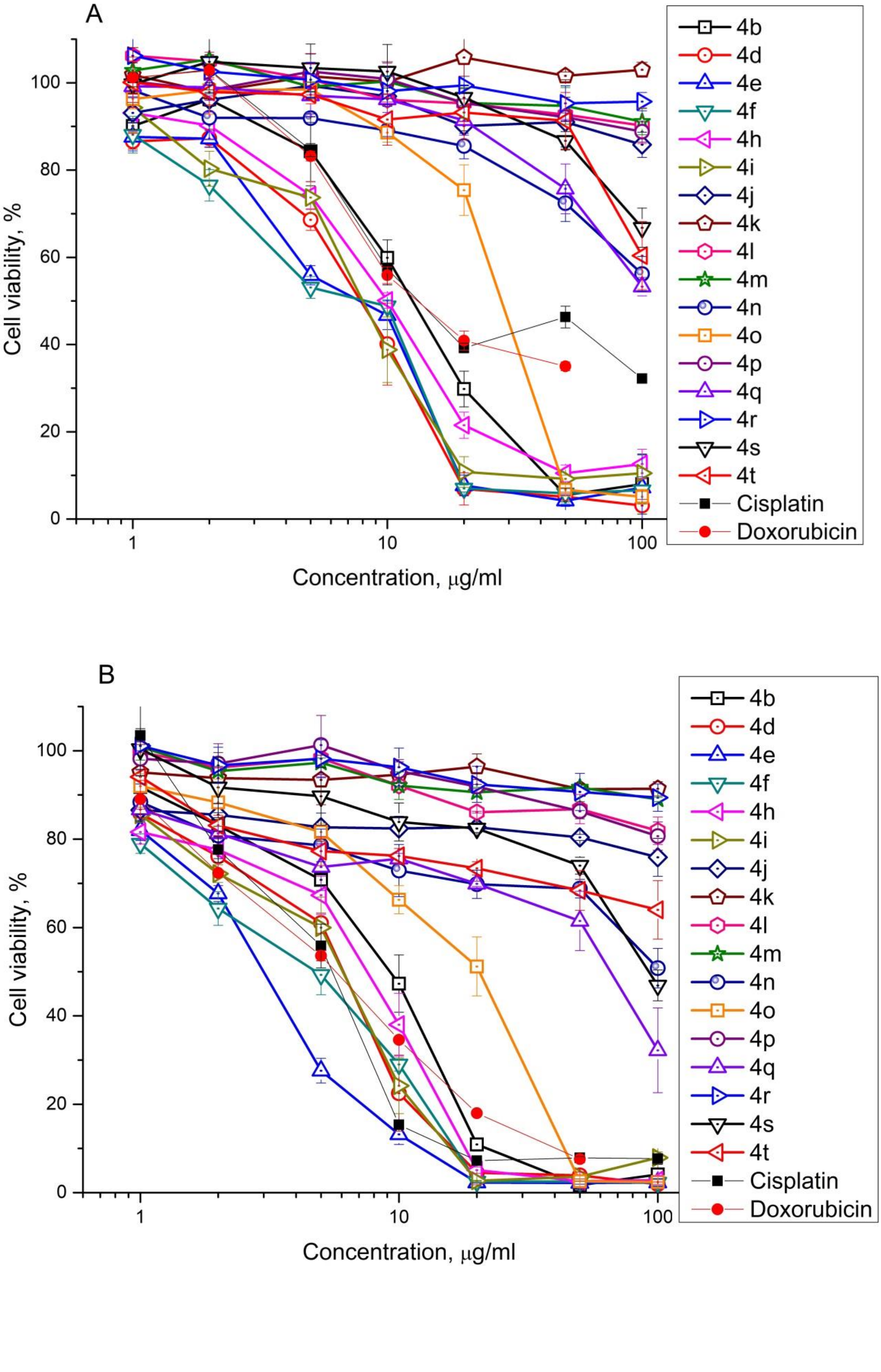

Figure S3. Cytotoxicity of 3-azaspiro[bicyclo[3.1.0]hexane-2,5′-pyrimidines] **4a-t** against CT26 cell line for 24 h (A) and 72 h (B).

2. In Silico Study

To predict the drug-likeness of selected compounds, the Lipinski rule of five was used. This rule describes molecular properties important for a drug's pharmacokinetics in a human body, including their absorption, distribution, metabolism and excretion (ADME). The Lipinski rule of five deals with simple physicochemical parameter ranges: Molecular weight (Mol. wt) ≤ 500, Octanol/water partition coefficient (C Log P) ≤ 5, H-bond donors ≤ 5 and H-bond acceptors ≤ 10. These parameters were calculated using Molinspiration online tool (https://www.molinspiration.com/cgi-bin/properties). Other ADME parameters such as Colon adenocarcinoma Caco2 permeability, Human intestinal absorption (HIA), Plasma protein binding ability (PPB) and ability to penetrate Blood–Brain-Barrier (BBB) were calculated using PREADMET online tool (https://preadmet.bmdrc.kr).

Table S1. Physicochemical properties of the synthesized compounds.

Compound	Mol. wt [1]	H-bond donors [2]	H-bond acceptors [3]	C Log P [4]	TPSA (Å2) [5]
Rule	⩽ 500	⩽ 5	⩽ 10	⩽ 5	⩽ 140
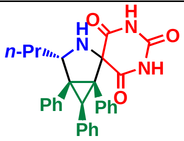 4d	465.55	3	6	4.17	87.29
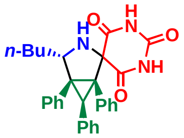 4e	479.58	3	6	4.67	87.29
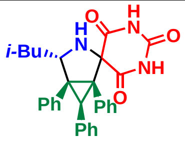 4f	479.58	3	6	4.38	87.29
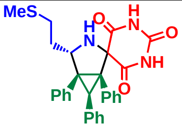 4h	497.62	3	6	3.52	87.29
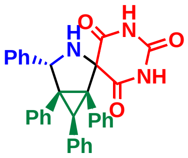 4i	499.57	3	6	4.32	87.29
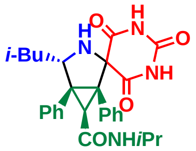 4t	502.62	4	8	3.51	116.39
Cisplatin	300,05	6	2	-4,58	55.28

[1] Molecular weight, [2] Number of hydrogen bond donors, [3] Number of hydrogen bond acceptors, [4] Logarithmic ratio of the octanol–water partitioning coefficient (C Log P), [5] Topological polar surface area (TPSA).

Table S2. Prediction of pharmacokinetic properties of the synthesized compounds.

Compound	Caco2 [1] permeability	HIA [2] (%)	PPB [3] (%)	BBB [4] (C_{brain}/C_{blood})
Rule	≥4	≥70	≥90	≥0.4
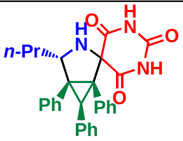 4d	20.9716	94.275941	99.621375	1.70696
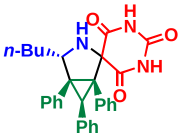 4e	21.0369	94.465005	99.489784	1.72804
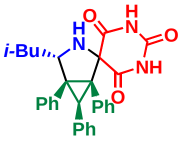 4f	21.1223	94.465238	99.000962	2.03649
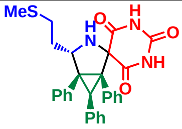 4h	21.094	94.787547	99.967144	0.223648
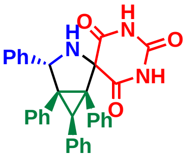 4i	21.102	95.300067	100.000000	1.42975
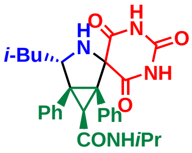 4t	21.1189	89.977411	85.329341	0.357404

[1] Colon adenocarcinoma permeability, [2] Human intestinal absorption, [3] Plasma protein binding,
[4] Blood–brain barrier penetration.
